# Calibration of an automated California mastitis test with focus on the device-dependent variation

**DOI:** 10.1186/2193-1801-3-760

**Published:** 2014-12-22

**Authors:** Anne-Christin Neitzel, Eckhard Stamer, Wolfgang Junge, Georg Thaller

**Affiliations:** Institute of Animal Breeding and Husbandry, Christian-Albrechts-University, D-24098 Kiel, Germany; TiDa Tier und Daten GmbH, D-24259 Westensee, Germany

**Keywords:** Automated California mastitis test, On-line somatic cell count, Calibration

## Abstract

The aim of the paper was to estimate the accuracy of the metrology of an installed indirect on-line sensor system based on the automated California Mastitis Test (CMT) with focus on the prior established device-dependent variation. A sensor calibration was implemented. Therefore, seven sensors were tested with similar trials on the dairy research farm Karkendamm (Germany) on two days in July 2011 and January 2012. Thereby, 18 mixed milk samples from serial dilutions were fourfold recorded at every sensor. For the validation, independent sensor records with corresponding lab somatic cell score records (LSCS) in the period between both trials were used (n = 1,357). From these records for each sensor a polynomial regression function was calculated. The predicted SCS (PSCS) was obtained for each sensor with the previously determined regression coefficients. Pearson correlation coefficients between PSCS and LSCS were established for each sensor and ranged between r = 0.57 and r = 0.67. Comparing the results with the correlation coefficients between the on-line SCS (OSCS) and the LSCS (r = 0.20 to 0.57) for every sensor, the calibration showed the tendency to improve the installed sensor system.

## Background

Mastitis is still the most costly and defiant disease for the dairy industry (Dadpasand et al. 2012). In German dairy herds, this disease is the second main reason for early culling after fertility problems. Annual statistics published by the German Cattle Breeders’ Federation (Bonn, Germany) showed that the percentage of culling because of udder diseases among all culling in German dairy herds was 14.9% in the year 2012 (ADR 2013). Mastitis has been the first focus of sensor developments, because of its major relevance for the dairy industry (Hogeveen et al. 2010).

The cow-level lab somatic cell count (LSCC), generally log-transformed to lab somatic cell score (LSCS), is the commonly used indirect trait for monitoring udder health for performance recording and genetic evaluation in Germany (Stampa et al. 2006). LSCC records are usually recorded monthly by the local herd recording organization and provide an important source of information for both, breeding and herd management. A potential shortcoming of the monthly LSCS as an indicator trait is, that acute short-duration infections may be difficult to identify simply from increased SCC during lactation (Urioste et al. 2010). Therefore, dairy producers have the possibility to use sensor systems for daily automatic on-line mastitis detection in composite or quarter milking (Koeck et al. 2012). Brandt et al. (2010) mentioned that sensors for on-farm analysis of milk composition are developed either for replacing visual inspection of foremilk by the milker (colour and image sensors) or for monitoring indicators in milk that have a high informational value but are not recognizable directly by the milker. Any ‘on-line’ mastitis detection is currently performed using electrical conductivity (EC), somatic cell count (SCC) or colour determination (Viguier et al. 2009), but the measurement of EC and SCC are the most common methods for mastitis detection in up-to-date milking systems (Brandt et al. 2010).

In the present study, the automated on-line SCC (OSCC) sensor system CellSense™ (Sensortec, Hamilton/Dairy Automation Limited, Waikato, New Zealand) was examined for its accuracy of metrology. The measuring principle based on the automated California Mastitis Test (CMT). The viscosity of a formed gel – milk sample mixed with a detergent-based chemical reagent – is called drain time (Whyte et al. 2004). Some of the fundamental chemical and rheological aspects of this gel formation were studied for developing a reliable parameter for early mastitis detection (Whyte et al. 2005; Verbeek et al. 2008). Selection decisions and bulk tank SCC management on dairy farms might be possible by identifying cows with udder health problems (Whyte et al. 2004).

Given that the OSCC is obtained from the drain time by each sensor using a specific algorithm, the sensor records might be comparable to the LSCC. Previous studies dealing with the mentioned sensor system analysed the correlation between lab and sensor. The manufacturers tested and calibrated one CellSense™ unit in the lab and on-line in an automatic milking system (AMS). Overall 83% (n = 197) of records in the calibration data set and 96% (n = 66) of data from on-line testing were correctly classified by the sensor (Whyte et al. 2004). Regarding their calibration approach, the manufacturers constituted a calibration curve for the OSCC sensor prototype (lab testing only), showing the location of the OSCC band boundaries (Whyte et al. 2002). The calibration curve was diagrammed by endorsing the records and charting the curve. Furthermore, Leslie et al. (2007) and Kamphuis et al. (2008) obtained correlation coefficients between lab and sensor records of r = 0.71 and r = 0.76 using only one CellSense™ unit.

In order to estimate the variations between seven different sensors and to use a more statistical calibration approach, the objectives of the current study were (1) to calculate a calibration curve with polynomial regression functions for each sensor with the trial data from July 2011 and January 2012, (2) to validate the sensor calibration with on-line sensor records in the period between both trials, (3) to obtain a predicted SCS (PSCS) for each sensor with prior determined regression coefficients and (4) to establish correlation coefficients between PSCS and LSCS for each sensor and to compare these results with the correlation coefficients between on-line SCS (OSCS) and LSCS.

## Material and methods

### Sensor data

Milk viscosity data were recorded routinely at the dairy research farm Karkendamm of the Institute of Animal Breeding and Husbandry, Christian-Albrechts-University in Kiel (Germany) between April 2011 and December 2012. Approximately 165 Holstein-Friesian dairy cows were milked twice daily in a rotary milking parlour on 28 milking stalls (GEA Farm Technologies). Dairy cows were classified into lactation number 1, 2 and ≥ 3. Forty-four lactation weeks were obtained. Automatic viscosity sensors were attached at the main milk-line on every fourth milking stall as an on-line system. The measuring principle based on the automated California Mastitis Test (CMT), which was developed in 1957 (Schalm and Noorlander 1957) and derived from the Whiteside test (Whiteside 1939). It is a common cow side test, especially used to identify subclinical mastitis (Leach et al. 2008). Data were available approximately two minutes after milk flow started. The viscosity of a formed gel is called drain time. It is the time the gel needs to flow through a standardized bore. From the drain time the OSCC of the milk sample is obtained using a specific algorithm. The OSCC was log-transformed to the OSCS as suggested by Ali and Shook (1980) to obtain nearly normal distribution. Drain time records included in the data were between 0.80 and 6.00 seconds. For further analysis, the drain time was log10(+1)-transformed (logDT). Cows which were milked into buckets were not assessed by the sensors.

### Sensor testing and mode of operation

For the calibration, sensors were tested in July 2011 and January 2012 with two similar experimental setups. High SCC milk (>2 mio. cells/ml) and low SCC milk (<200,000 cells/ml) was obtained from two randomly selected cows in the research dairy herd and combined in various proportions within a serial dilution as implemented by Whyte et al. (2004). Therefore, 18 mixed milk samples (nine mixed milk samples per experimental setup) were fourfold tested at each of the seven sensors. For on-line testing, the 18 mixed milk samples were fourfold tested by connecting plastic syringes – filled with 60 ml of each mixed milk sample inside – with the sampling well-tube of each sensor. While the milking rotary rotated slowly, one sensor after another received a milk start signal (starting with sensor no. 1) and started automatic sampling after approximately 35 seconds. Sampling, mixing, measuring and cleaning was performed according to Whyte et al. (2004) and was completed automatically. When sensor no. 1 arrived after one entirely turn of the rotary parlour, the start button was pressed two times and the next measuring procedure started. This was repeated four times for every sensor. Therefore, 72 measurement data for each sensor and altogether 504 measurement data for all these seven sensors were obtained.

The seven automatic viscosity sensors were attached on every fourth milking stall as an on-line system and data were obtained for one out of four cows at each milking. Hence, for the sensor validation (verification of sensor calibration), the under practical conditions routinely recorded on-line sensor data between both experimental setups in the period between July 2011 to January 2012 (n = 1,357) with the corresponding LSCS were consulted.

### Data from herd management system and laboratory

Milk yield was automatically recorded at every milking in the herd management system (DairyPlan, GEA Farm Technologies). Milk composition was analysed weekly based on samples collected from two consecutive milkings. For the present research, two reference samples of each mixed milk sample in both trials were taken (n = 36) and submitted to the laboratory of the local dairy herd recording organisation for LSCC determination. The two LSCC records of each mixed milk sample were averaged for further examinations (n = 18). According to Kamphuis et al. (2008), the LSCC was regarded as the reference. For further analysis the LSCC was log-transformed to the LSCS as suggested by Ali and Shook (1980). Only LSCS information was considered in further analysis.

### Statistical analysis

The SAS package (SAS® 2010) was used for descriptive and statistical analyses. Statistical significance was defined at P ≤ 0.05. The distributions of LSCC and drain time were tested using the UNIVARIATE procedure (SAS® 2010). In Table 1, the analysed descriptive statistics of sensor and laboratory records for the calibration data set (n = 504) and the validation data set (n = 1,357) are presented. A threshold OSCC value of 0 was possible, because Whyte et al. (2004) accounted a need of farmers for flexible band reporting, rather than strictly quantitative results. For the parameter OSCS, a lower observation number was used in the calibration data set (n = 478) and the validation data set (n = 310), because the log-transformation of a value of 0 resulted in a missing value (Table 1).

**Table 1:** **Descriptive statistics: number of observations (N), median, mean value, standard deviation (SD), minimal (Min) and maximal (Max) values for the sensor and laboratory determined records in the calibration and the validation data set**

Indicator^1^	Calibration data set						Validation data set					
	N	Median	Mean	SD	Min	Max	N	Median	Mean	SD	Min	Max
CellSense™												
DT	504	1.94	2.01	0.36	1.33	3.67	1,357	1.44	1.52	0.28	0.92	5.29
logDT	504	1.29	1.30	0.07	1.13	1.56	1,357	1.16	1.18	0.06	0.97	1.72
OSCC	504	364	437	358	0	2,111	1,357	0	67	226	0	3,760
OSCS	478	2.58	2.55	0.35	0.78	3.33	310	3.72	3.65	1.71	-2.06	8.23
Laboratory												
LSCC	504	679	855	641	45	2,597	1,357	56	164	411	5	6,320
LSCS	504	5.76	5.70	1.22	1.85	7.70	1,357	2.16	2.45	1.66	-1.32	8.89

^1^DT = drain time (sec); logDT = log-transformed drain time (log10(DT) + 1); OSCC = on-line somatic cell count (1000/ml); OSCS = on-line somatic cell score (log2(OSCC/100) + 3); LSCC = laboratory somatic cell count (1000/ml); LSCS = laboratory somatic cell score (log2(LSCC/100) + 3).

For the estimation of the residuals and residual variances for each sensor a linear mixed model was used, which was as follows:

$$\begin{array}{c} y_{\text{ijklm}}=\;\mu +{SE}_{i}+\;{td}_{j}+td*{\text{ms}}_{k}+td*\text{ms}*{\mathrm{sr}}_{l}\\ \;+\;e_{\text{ijklm}} \end{array}$$

where y_ijklm_ = observation of logDT, μ = overall mean, SE_i_ = fixed effect of the i^th^ sensor (i = 1 to 7), td_j_ = random effect of the j^th^ test day (j = 1 to 2), ms_k_ = within test day nested random effect of the k^th^ mixed milk sample (k = 1 to 18), sr_l_ = within test day and mixed milk sample nested random effect of the l^th^ sample run (l = 1 to 4) and e_ijklm_ = random residual effect of the ijklm^th^ observation.

Assuming heteroscedasticity instead of homoscedasticity in the linear mixed model, a residual variance was estimated for each sensor. Thereby, the device-dependent variation was determined. The sensors were sorted by magnitude of the residual variance.

The partial least square regression (PLSR) statistical procedure was applied to the trial data (Pullanagari 2011). Linear and polynomial regression of the records in the calibration data set was compared. Regarding the adjusted coefficient of determination (R^2^), the polynomial regression was chosen for further examination. Therefore, the RSREG procedure in the SAS package (SAS® 2010) was performed in order to establish relationships between lab estimated values and the values predicted from calibration equations. The method of least squares was used to fit quadratic response surface regression models (SAS® 2010). The regression coefficients were obtained by polynomial regression of X versus Y in the calibration process (Hansen and Schjoerring 2003). Differences in coefficients of determination (R^2^) and root mean square errors (RMSE) were compared to test the performance of the calibration models (Pullanagari 2011).

For external validation, the under practical conditions obtained sensor records of the twice daily milking between both tests with corresponding lab information were used. The predicted SCS (PSCS) was calculated for each sensor with the previously obtained regression models using the validation data set. The Pearson correlation coefficients between PSCS and LSCS for each sensor were determined. Compared with the correlations between PSCS and OSCS for each sensor, the quality of the predicted variable PSCS and the accuracy of the sensor calibration were validated.

## Results and discussion

The results of the linear mixed model showed, that the variation between the sensors is significant (P < 0.0001). For the clarification of potential differences between the sensor effects, the residuals were calculated for each sensor with regard to the random effects observed in the linear mixed model. The results offered a high significant device-dependent variation (P < 0.0001). Residual variances ranged between 0.000106 (log SCC/ml) and 0.000814 (log SCC/ml).

Consequently, a further estimation of the variations between sensor and lab records was required. Pearson correlation coefficients between sensor and lab data were illustrated for the calibration data set in Table 2. High correlations were found between drain time and LSCC (r = 0.75) and logDT and LSCS (r = 0.79). The correlation between OSCC and LSCC was r = 0.91 (P < 0.0001). The OSCS was well correlated (r = 0.80) with the LSCS.

**Table 2:** **Pearson correlation coefficients between sensor records and laboratory records calculated with data from the calibration data set**

Parameter^1^	DT	logDT	OSCC	OSCS
OSCS	-	0.87	-	-
LSCC	-	-	0.91	-
LSCS	0.75	0.79	-	0.80

P < 0.0001; ^1^DT = drain time (sensor determined; sec); logDT = log-transformed drain time (log10(DT) + 1); OSCC = on-line somatic cell count (sensor determined; 1000/ml); OSCS = on-line somatic cell score (log2(OSCC/100) + 3); LSCC = somatic cell count (laboratory determined; 1000/ml); LSCS = somatic cell score (laboratory determined; log2(LSCC/100) + 3).

Few studies dealing with the mentioned sensor system analysed the correlation between lab and sensor. Leslie et al. (2007) evaluated the diagnostic test characteristics of one CellSense™ unit against lab records. The correlation between OSCC and LSCC was r = 0.71, which was obvious lower than the correlation found in this study (r = 0.91). A CellSense™ unit was also tested by Kamphuis et al. (2008) in an AMS. They reported more variation in OSCC values at lower values of LSCC, meaning that a more linear relationship was found between OSCC and LSCC at greater values of LSCC. This was reflected in their calculated correlation coefficients between OSCS and LSCS with r = 0.76 (P < 0.001), which mostly provided present findings. In comparison to the correlations mentioned in the literature, the results of the present study showed higher correlation coefficients. Regarding quarter level SCC data recorded with these sensor system installed in an AMS, Mollenhorst et al. (2009) found a correlation coefficient of r = 0.47 (P < 0.001) between laboratory determined quarter SCS and OSCS, with an increasing correlation at higher SCC values.

Nevertheless, the estimated variations between the seven sensors should lead to a more statistical calibration approach compared with Whyte et al. (2004). Therefore, calibration curves were calculated with polynomial regression functions for each sensor with the trial data from July 2011 and January 2012. As the trait of interest, the accuracy of measurement was tested. Therefore the coefficients of determination (R^2^) and root mean square errors (RMSE) for the seven sensors were presented in Table 3. The results showed large differences between the sensors (R^2^ = 56.1 to 87.2%) with good accuracy (RMSE = 0.82 to 0.44 (log SCC/ml)).

**Table 3:** **Differences of the coefficient of determination (R** ^**2**^ **) and the root mean square error (RMSE) of the polynomial regression models for each sensor calculated with data from the calibration data set**

Parameter^1^	Sensor						
	1	2	3	4	5	6	7
R^2^	87.2	60.3	56.1	77.5	71.4	76.2	81.5
RMSE	0.44	0.78	0.82	0.59	0.66	0.60	0.53

^1^R^2^ (%); RMSE (log SCC/ml).

The sensor calibration was validated with on-line sensor records in the period between both trials and a PSCS was obtained for each sensor with prior determined regression coefficients. Table 4 showed the correlation coefficients for the sensors between LSCS and PSCS, determined with data from the validation data set. According to expectations, the correlation coefficients for the validation data set were lower compared with those of the calibration data set, conscious that for OSCS a lower observation number was used. The correlations between OSCS and LSCS ranged between r = 0.20 and r = 0.57, whereas correlations between PSCS and LSCS varied from r = 0.57 to 0.67 (P < 0.0001).

**Table 4:** **Pearson correlation coefficients for the installed seven sensors between laboratory somatic cell score (LSCS) and predicted somatic cell score (PSCS) determined with data from the validation data set, and the differences (∆) between the correlation coefficients as the quality of the calibration approach**

Sensor	Parameter^1^	OSCS	LSCS	∆
1	LSCS	0.57	-	0.10
	PSCS	-	0.67	
2	LSCS	0.22	-	0.40
	PSCS	-	0.62	
3	LSCS	0.33	-	0.33
	PSCS	-	0.66	
4	LSCS	0.20	-	0.37
	PSCS	-	0.57	
5	LSCS	0.49	-	0.13
	PSCS	-	0.62	
6	LSCS	0.51	-	0.10
	PSCS	-	0.61	
7	LSCS	0.26	-	0.34
	PSCS	-	0.60	

P < 0.0001; ^1^OSCS = on-line somatic cell score (log2(OSCC/100) + 3); LSCS = somatic cell score (laboratory determined; log2(LSCC/100) + 3); PSCS = predicted somatic cell score; ∆ = IdifferenceI.

Whyte et al. (2004) tested and calibrated a CellSense™ unit in the lab and on-line in an AMS. Regarding their calibration approach, the inventors constituted a calibration curve for the OSCC sensor prototype, which was diagrammed by endorsing the records and charting the curve. Overall 238 laboratory samples and 69 samples in the field were tested. The used SCC thresholds of the sensor system were outlined in a five-band scale: < 200; 200 to 500; 500 to 1,500; 1,500 to 5,000 and > 5,000 (1,000 cells/ml). During laboratory testing, the sensor correctly detected 95%, 85%, 76%, 72% and 95% of samples in each of the five mentioned bands. Overall 83% (n = 197) of records in the calibration data set and 96% (n = 66) of data from on-line testing were correctly classified by the sensor (Whyte et al. 2004).

A potential explanation for the moderate correlations between PSCS and LSCS might be the influence of milk sample density and composition. It cannot be excluded, that milk components (e.g. fat or protein content) might have an effect on the sensor data, but that could not be surveyed with the underlying test results. In this study, the sample components were self-provided in the mixed milk samples within the serial dilution, because no standardisation for milk ingredients was realised. Therefore, those milk components were not consulted for further examination. Schalm and Noorlander (1957) mentioned that the used reagent for the CMT was chosen, because it did not involve the milk fat as part of the visible positive reaction with mastitic milk. Nevertheless, the effect of other milk components on the sensor records must be analysed in further studies.

It should also be mentioned, that the sensor system and the lab use different measuring principles. The sensor system in this study used the automated CMT, where a milk sample is mixed with the detergent-based chemical reagent and the viscosity from the gel is indirectly obtained (Whyte et al. 2005). On the other hand, the lab used the directly fluoro-optic electronic cell counting method by disk cytometry (e.g. Fossomatic 5000, Foss Electric, Hillerød), where the cell nuclei are stained (e.g. with ethidium bromide) and counted by light scatter, or fluorescent detectors, or both (Brandt et al. 2010).

The accuracy of the sensor calibration was validated by comparing the differences (∆) between the previous determined correlation coefficients between OSCS and LSCS (r = 0.20 to 0.57) with those between PSCS and LSCS (r = 0.57 to 0.67) (Table 4). The correlations examined for the predicted records achieved higher values for each sensor. Hence, the differences ranged between ∆ = 0.10 and 0.40. Therefore, the validation of the calibration models indicated that the calibration approach in this study showed the tendency to improve the on-line sensor system sustainable.

## Conclusions

The indirect on-line sensor system based on the automated California Mastitis Test was calibrated with a more statistical calibration approach as compared with the calibration strategy of the manufacturers. The calibration showed the tendency to improve the sensor system. Nevertheless, the robustness of the sensor system is not specified so far, due to the sensor calibration method which was only executed for the on-line installed sensor system in the present study.

## Abbreviations

CMT: California mastitis test
LSCC: Laboratory somatic cell count
LSCS: Laboratory somatic cell score
PSCS: Predicted somatic cell score
OSCC: On-line somatic cell count
OSCS: On-line somatic cell score
logDT: Log-transformed drain time
PLSR: Partial least square regression
RMSE: Root mean square errors.
